# Distinct biological and molecular characteristics of breast cancer in young women: a narrative review

**DOI:** 10.1097/MS9.0000000000004896

**Published:** 2026-03-23

**Authors:** Emmanuel Ifeanyi Obeagu

**Affiliations:** a Division of Haematology, Department of Biomedical and Laboratory Science, Africa University, Zimbabwe; b The Department of Molecular Medicine and Haematology, School of Pathology, Faculty of Health Sciences, University of the Witwatersrand, Johannesburg, South Africa

**Keywords:** breast cancer, hormone receptors, molecular profiling, tumor biology, young women

## Abstract

Breast cancer in young women (defined as ≤40 years of age) presents unique clinical and biological challenges. While it accounts for a small proportion of total breast cancer cases, it is often associated with more aggressive tumor phenotypes, higher-grade histology, and advanced-stage diagnosis. Young patients are more likely to develop triple-negative and HER2-positive subtypes, which are linked to poor prognostic outcomes and limited treatment options. These clinical patterns suggest that age-specific molecular mechanisms drive tumor behavior in this population. Emerging molecular profiling studies have revealed distinct genomic, transcriptomic, and epigenetic features in breast cancer arising in young women. Mutations in TP53, BRCA1/2, and other DNA repair genes are more prevalent, along with basal-like intrinsic subtypes that exhibit high proliferation and genomic instability. The tumor microenvironment also demonstrates unique immune signatures, including increased tumor-infiltrating lymphocytes and inflammatory cytokine expression, which may influence response to immunotherapy and tumor progression. This review synthesizes current knowledge on the unique tumor biology of breast cancer in young women, highlighting the need for precision oncology approaches that account for age-related tumor behavior. Enhanced characterization of this population may ultimately improve survival outcomes and quality of life for young women affected by breast cancer.

## Introduction

Breast cancer remains the most common malignancy among women globally and a leading cause of cancer-related mortality. While the majority of breast cancer cases occur in postmenopausal women, approximately 5–7% are diagnosed in women aged 40 years or younger. This subset, often referred to as “early-onset” or “young women’s breast cancer,” poses distinct clinical, biological, and psychosocial challenges. Notably, young women frequently present with advanced-stage disease, high-grade tumors, and a greater risk of recurrence, despite receiving aggressive treatment regimens^[^1^]^. Epidemiological trends suggest that the incidence of breast cancer in young women is gradually increasing in some regions, particularly in low- and middle-income countries. This may be attributed to shifts in reproductive behavior, genetic predispositions, environmental exposures, and increased awareness leading to earlier detection. However, younger patients often lack identifiable risk factors and are less likely to undergo routine screening, which contributes to delayed diagnoses and poorer outcomes^[^2^]^.

HIGHLIGHTS

- Breast cancer in young women is biologically aggressive and often presents at advanced stages.
- Higher prevalence of TNBC and HER2-positive subtypes.
- Enriched for BRCA1/2 and TP53 mutations.
- Distinct tumor microenvironment with increased TILs.
- Age-specific management, including tailored systemic therapy and fertility considerations, is crucial for improved outcomes.

Clinically, breast cancer in young women tends to exhibit unfavorable characteristics, including larger tumor size at diagnosis, nodal involvement, and higher rates of lymphovascular invasion. More importantly, the disease is biologically distinct, with a higher prevalence of aggressive subtypes such as triple-negative breast cancer (TNBC) and Human Epidermal Growth Factor Receptor 2 (HER2)-enriched tumors. These molecular subtypes are known for their rapid progression, limited therapeutic options, and reduced overall survival compared to hormone receptor-positive tumors more common in older women^[^3,4^]^. At the molecular level, young women’s breast tumors demonstrate unique genetic and epigenetic features. Germline mutations in BRCA1 and BRCA2 are disproportionately represented in this age group, with associated basal-like phenotypes. Somatic mutations in TP53, PIK3CA, and other oncogenic drivers are also more frequently observed, suggesting a distinct tumorigenic pathway. Furthermore, transcriptomic analyses have revealed differences in gene expression patterns related to cell proliferation, DNA damage response, and immune signaling^[^5^]^. The tumor microenvironment in younger patients may further contribute to disease aggressiveness. Emerging studies suggest that tumors in young women display enhanced angiogenic activity, increased immune cell infiltration, and elevated levels of pro-inflammatory cytokines. These alterations can influence tumor growth, metastatic potential, and response to therapy^[^6^]^.

### Aim

This review aims to provide a comprehensive synthesis of current research on the unique biological and molecular characteristics of breast cancer in young women. It seeks to elucidate the distinct tumor biology, genomic and epigenomic alterations, hormone receptor and HER2 expression patterns, and tumor microenvironment features that contribute to the aggressive clinical behavior observed in this population.

## Methods

This narrative review was conducted through a comprehensive and systematic search of the existing literature to synthesize current knowledge on the biological and molecular characteristics of breast cancer in young women. Multiple electronic databases, including PubMed, Scopus, and Web of Science, were searched for relevant peer-reviewed articles published in English from 2000 to 2025. Keywords and Medical Subject Headings (MeSH) terms used included combinations of “breast cancer,” “young women,” “tumor biology,” “molecular profiles,” “genomics,” “epigenetics,” “hormone receptor,” “HER2,” and “tumor microenvironment.”

Eligible studies comprised original research articles, reviews, meta-analyses, and clinical trials focusing on the unique molecular and biological features of breast cancer in women aged 40 years and younger. Articles addressing general breast cancer biology without age stratification were excluded unless they provided relevant data or insights applicable to younger populations. Additional references were identified through citation tracking and manual review of bibliographies of selected articles. Data from included studies were qualitatively synthesized to capture key themes regarding hormone receptor and HER2 expression patterns, genomic and epigenomic alterations, tumor microenvironment dynamics, molecular pathways, and clinical implications. The review emphasizes findings that contribute to understanding the distinct disease biology in young women and its impact on diagnosis, treatment, and outcomes.

### Hormone receptor and HER2 expression patterns

Hormone receptor (HR) and HER2 status remain critical determinants of breast cancer classification, prognosis, and treatment. In young women with breast cancer, distinct patterns of hormone receptor and HER2 expression have been consistently observed, setting this population apart from older patients in terms of tumor biology and clinical behavior^[^7^]^. Compared to postmenopausal women, young women are more likely to be diagnosed with hormone receptor-negative [estrogen receptor (ER) and/or progesterone receptor (PR) negative] tumors. Studies have shown a disproportionately high prevalence of TNBC in this age group – a subtype defined by the absence of ER, PR, and HER2 expression. TNBC is particularly concerning due to its aggressive clinical course, higher rates of distant metastasis, and lack of targeted hormonal or HER2-directed therapies. Additionally, these tumors tend to exhibit basal-like features on molecular profiling, often associated with BRCA1 mutations^[^8^]^.

Conversely, when hormone receptor-positive tumors do occur in young women, they often display higher proliferative indices, such as increased Ki-67 expression, and reduced expression of progesterone receptors. This suggests that even HR-positive tumors in this demographic may behave more aggressively than similar tumors in older patients. Furthermore, premenopausal hormonal fluctuations and the influence of ovarian estrogen production may interact with tumor biology in ways not yet fully elucidated^[^9^]^. HER2 overexpression or gene amplification is also more frequently observed in young women compared to older counterparts. HER2-positive tumors tend to be highly proliferative and may present at a more advanced stage. However, the advent of HER2-targeted therapies, such as trastuzumab and pertuzumab, has significantly improved outcomes in this subgroup. The high prevalence of HER2 positivity in younger patients underscores the importance of timely HER2 testing and access to targeted treatments^[^10^]^. Importantly, molecular subtyping using platforms like PAM50 has confirmed the dominance of more aggressive intrinsic subtypes – basal-like and HER2-enriched – in young women. In contrast, the luminal A subtype, typically characterized by hormone receptor positivity and lower proliferation rates, is less common in this population. These differences have therapeutic implications, as luminal A tumors often respond well to endocrine therapy alone, while more aggressive subtypes may require multi-modal treatment approaches including chemotherapy and biologics^[^11^]^.

### Genomic and epigenomic signatures in breast cancer in young women

The molecular landscape of breast cancer in young women is increasingly recognized as distinct from that of older patients, marked by unique genomic alterations and epigenetic modifications that influence tumor behavior, treatment response, and clinical outcomes. These differences suggest that breast cancer in young women may arise through alternative oncogenic pathways, warranting a deeper investigation into their molecular underpinnings^[^12^]^. Genomic analyses have identified a higher prevalence of germline mutations in young breast cancer patients, particularly in the BRCA1 and BRCA2 genes. BRCA1-mutated tumors in this group often exhibit a basal-like phenotype, characterized by triple-negative receptor status, high proliferative activity, and genomic instability. Beyond BRCA mutations, young women with breast cancer frequently harbor somatic alterations in TP53, a tumor suppressor gene associated with poor prognosis, and PIK3CA, a key player in the PI3K/AKT/mTOR pathway. Alterations in DNA repair genes, chromatin remodeling genes, and cell-cycle regulators are also more commonly seen in this age group, underscoring a heightened reliance on pathways related to genomic maintenance and cellular proliferation^[^13^]^.

In addition to genetic mutations, epigenetic dysregulation plays a critical role in shaping the molecular phenotype of breast cancer in young women. DNA methylation profiling has revealed distinct methylation signatures in early-onset breast tumors, often involving genes related to cell adhesion, immune signaling, and hormone receptor expression. For example, hypermethylation of the ESR1 promoter region may contribute to reduced estrogen receptor expression in some HR-negative tumors. Aberrant methylation patterns have also been implicated in treatment resistance, particularly in the context of chemotherapy and endocrine therapy^[^14^]^. Histone modifications and non-coding RNAs, including microRNAs (miRNAs) and long non-coding RNAs (lncRNAs), further contribute to the complex regulatory networks governing tumor biology in young women. Certain miRNAs – such as miR-21, miR-155, and miR-221 – are frequently dysregulated and have been linked to increased invasiveness, angiogenesis, and immune evasion. These epigenetic modulators may serve as both biomarkers of disease aggressiveness and potential therapeutic targets^[^15^]^. Emerging multi-omics studies that integrate genomic, transcriptomic, and epigenomic data provide a more comprehensive view of the molecular heterogeneity within young women’s breast cancers. These analyses reinforce the notion that breast cancer in young women is not merely a chronological variant of the disease but a biologically unique entity with distinct evolutionary pressures and oncogenic drivers (Table 1)^[^16^]^.

**Table 1:** Genomic and epigenomic signatures in breast cancer in young women.

Alteration Type	Specific Genes or Features	Frequency/Prevalence	Functional Impact	Clinical Relevance
**Germline mutations**	*BRCA1, BRCA2, TP53, PALB2*	High in hereditary cases	Deficient DNA repair, genomic instability	Predicts PARP inhibitor sensitivity; guides genetic counseling
**Somatic mutations**	*PIK3CA, TP53, GATA3, MYC, RB1*	Variable by subtype	Alters cell cycle, proliferation, apoptosis	May inform targeted therapy and prognosis
**Gene amplifications**	*MYC, CCND1, HER2 (ERBB2)*	More common in aggressive subtypes	Drives oncogenesis and proliferation	Targetable with HER2- or CDK4/6-inhibitors
**Epigenetic silencing**	*BRCA1, CDH1, RASSF1A* (via promoter methylation)	Frequently observed	Loss of tumor suppressor gene expression	May inform response to demethylating agents or epigenetic drugs
**miRNA dysregulation**	miR-21, miR-155 (up); miR-34a, miR-200c (down)	Frequently reported in young women	Modulates gene expression, EMT, and immune evasion	Potential biomarkers; targets for future therapeutics
**Chromatin remodeling**	Mutations in *ARID1A, KMT2C, EZH2*	Less frequent, subtype-dependent	Affects transcriptional regulation and cell fate	Emerging targets for epigenetic therapy
**Methylation profiles**	Hypermethylation of ER-related genes	More prominent in ER-negative cancers	Affects hormonal signaling pathways	May influence endocrine therapy resistance

### Tumor microenvironment and immune landscape in breast cancer in young women

The tumor microenvironment (TME) plays a pivotal role in breast cancer development, progression, and response to therapy. In young women, accumulating evidence suggests that the TME exhibits distinct immunological and stromal characteristics that may contribute to the aggressive nature of their tumors^[^17^]^. Studies indicate that breast tumors in young women often present with elevated levels of tumor-infiltrating lymphocytes (TILs), particularly in triple-negative and HER2-positive subtypes. High TIL density has been associated with improved prognosis and enhanced response to chemotherapy and immunotherapy in these aggressive subtypes. However, despite this potentially favorable immune infiltration, the immune milieu in young women’s tumors may be functionally compromised due to immunosuppressive factors within the TME, including regulatory T cells (Tregs), myeloid-derived suppressor cells (MDSCs), and tumor-associated macrophages (TAMs) with pro-tumorigenic M2 phenotypes^[^18,19^]^.

Angiogenesis and stromal remodeling are also prominent features of the TME in young women’s breast cancer. Increased vascular endothelial growth factor (VEGF) expression and abnormal tumor vasculature contribute to enhanced tumor growth and metastasis. The dense and reactive stroma facilitates invasion and creates a hypoxic environment that promotes genomic instability and resistance to therapies. These stromal changes may be more pronounced in younger patients, potentially exacerbating the aggressive clinical behavior observed^[^20^]^. Cytokine and chemokine profiles within the TME further modulate tumor-immune interactions. Elevated levels of pro-inflammatory cytokines such as interleukin-6 (IL-6), tumor necrosis factor-alpha (TNF-α), and transforming growth factor-beta (TGF-β) have been documented in tumors from young women, fostering a microenvironment conducive to tumor progression, immune evasion, and metastasis. Additionally, the interplay between cancer-associated fibroblasts (CAFs) and immune cells influences extracellular matrix remodeling and immune cell recruitment, further shaping tumor dynamics^[^21^]^. The immune checkpoint landscape in breast cancer of young women is gaining research interest. Expression of programmed death-ligand 1 (PD-L1) is variably increased, particularly in TNBC, supporting the rationale for immune checkpoint inhibitors in this subgroup. However, responses to immunotherapy remain heterogeneous, highlighting the need for better predictive biomarkers and combination strategies to overcome resistance (Table 2)^[^22^]^.

**Table 2:** Tumor microenvironment and immune landscape in breast cancer in young women.

Component	Feature/Marker	Functional Role	Clinical Implication
**Tumor-Infiltrating Lymphocytes (TILs)**	CD8⁺ T cells, CD4⁺ T cells, B cells	Mediate anti-tumor immune response	High TILs predict better prognosis and response to chemotherapy
**Regulatory T cells (Tregs)**	FOXP3⁺	Suppress effector T-cell function	Associated with immune evasion and poor outcomes
**Myeloid-Derived Suppressor Cells (MDSCs)**	CD11b⁺, Gr1⁺ markers	Inhibit T-cell activity and promote tumor growth	Linked to immunosuppression and treatment resistance
**Tumor-Associated Macrophages (TAMs)**	M2 phenotype (CD163⁺, CD206⁺)	Promote angiogenesis, EMT, and metastasis	High M2 infiltration correlates with aggressive disease
**Cancer-Associated Fibroblasts (CAFs)**	FAP, α-SMA	Remodel ECM, promote invasion and immune modulation	Targetable component in stroma-directed therapies
**Angiogenic factors**	VEGF, ANGPT2	Drive neovascularization and tumor perfusion	VEGF overexpression linked to metastasis; anti-angiogenic therapy
**Cytokines and Chemokines**	IL-6, IL-8, TGF-β, TNF-α, CCL2	Modulate immune infiltration, inflammation, and tumor behavior	Potential biomarkers and immunotherapy targets
**Immune Checkpoint Molecules**	PD-L1, CTLA-4	Suppress anti-tumor immunity	PD-L1 expression supports use of immune checkpoint inhibitors
**Stromal Hypoxia**	HIF-1α, carbonic anhydrase IX	Enhances EMT, immune evasion, and therapy resistance	Contributes to poor prognosis and treatment failure
**Extracellular Matrix (ECM) Density**	Collagen I/III, fibronectin	Alters immune cell infiltration and facilitates metastasis	Indicator of aggressive stroma; potential imaging/therapeutic target

### Molecular pathways and targetable mechanisms in breast cancer in young women

Breast cancer in young women is characterized by the activation of multiple oncogenic molecular pathways that contribute to its aggressive clinical behavior and therapeutic challenges. Elucidation of these pathways has provided insight into the biological basis of early-onset breast cancer and revealed potential targets for precision therapies aimed at improving outcomes in this high-risk population^[^23^]^. One of the central pathways implicated in young women’s breast cancer is the PI3K**/**AKT**/**mTOR signaling cascade. Aberrations in this pathway, including activating mutations in PIK3CA and loss of PTEN function, promote cell proliferation, survival, and resistance to endocrine therapies. Given the pathway’s prominence, several inhibitors targeting PI3K or mTOR complexes are currently under clinical investigation and have shown promise, particularly in hormone receptor-positive tumors with resistant phenotypes^[^24^]^.

The DNA damage response (DDR) pathway also plays a critical role in breast cancer among young women, especially those harboring germline mutations in BRCA1, BRCA2, and other homologous recombination repair genes. Deficiencies in DDR render tumors sensitive to poly (ADP-ribose) polymerase (PARP) inhibitors, which exploit synthetic lethality to induce cancer cell death. PARP inhibitors have gained approval for BRCA-mutated breast cancers and are being actively evaluated in clinical trials for broader application in early-onset disease^[^25^]^. Aberrant activation of the epidermal growth factor receptor (EGFR) family, including HER2, represents another key driver in young women’s breast cancer. HER2 amplification and overexpression correlate with increased tumor aggressiveness but also provide an actionable target. HER2-directed therapies, such as trastuzumab, pertuzumab, and newer antibody-drug conjugates, have revolutionized treatment, markedly improving survival rates in HER2-positive young patients^[^26^]^.

Additional molecular pathways, including the NOTCH, WNT/β-catenin, and MAPK/ERK pathways, have been implicated in breast cancer progression in younger women. Dysregulation of these signaling networks contributes to stemness, epithelial-to-mesenchymal transition (EMT), and metastasis. Although targeted agents against these pathways remain largely experimental, they represent promising avenues for future therapeutic development^[^27^]^. Hormone receptor-positive tumors in young women also exhibit resistance mechanisms to endocrine therapy, often driven by cross-talk between estrogen receptor signaling and growth factor pathways. Understanding these complex interactions has informed combination strategies incorporating CDK4/6 inhibitors, which have demonstrated significant efficacy in improving progression-free survival in premenopausal patients (Table 3)^[^28^]^.

**Table 3:** Molecular pathways and targetable mechanisms in breast cancer in young women.

Pathway	Key components	Biological role	Targeted therapies	Clinical implications
**PI3K/AKT/mTOR**	PIK3CA, PTEN, AKT1, mTOR	Promotes cell growth, survival, and resistance	PI3K inhibitors (alpelisib), mTOR inhibitors (everolimus)	Frequently altered in young women; resistance to endocrine therapy
**DNA Damage Response (DDR)**	BRCA1, BRCA2, ATM, CHEK2	Maintains genomic stability; regulates DNA repair	PARP inhibitors (olaparib, talazoparib)	Germline mutations prevalent; synthetic lethality exploited therapeutically
**HER2/ERBB2 Signaling**	HER2 (ERBB2), HER3, EGFR	Stimulates proliferation and metastasis	Trastuzumab, pertuzumab, T-DM1, neratinib	High HER2 expression linked to aggressive tumors; strong response to anti-HER2 agents
**Estrogen Receptor (ER) Pathway**	ESR1, GATA3, FOXA1	Drives hormone-responsive tumor growth	SERMs (tamoxifen), aromatase inhibitors, CDK4/6 inhibitors	Common in young women but often shows therapy resistance
**MAPK/ERK Pathway**	RAS, RAF, MEK, ERK	Controls cell cycle, proliferation, and differentiation	MEK inhibitors (trametinib – experimental)	May contribute to endocrine resistance; combinable with ER-targeted agents
**NOTCH Signaling**	NOTCH1–4, DLL1, JAG1	Influences stemness and therapy resistance	Gamma-secretase inhibitors (under investigation)	Emerging target in basal-like and triple-negative subtypes
**WNT/β-catenin Pathway**	CTNNB1 (β-catenin), APC, WNT ligands	Regulates cell polarity, EMT, and immune evasion	WNT inhibitors (experimental)	Linked to tumor stemness and metastasis in early-onset cases
**Immune Checkpoints**	PD-1, PD-L1, CTLA-4	Suppresses T-cell–mediated tumor immunity	Immune checkpoint inhibitors (atezolizumab, pembrolizumab)	Potential benefit in TNBC and high-TIL tumors in young women
**CDK4/6 Cell Cycle Regulation**	CDK4, CDK6, RB1, cyclin D1	Governs G1/S phase transition and proliferation	CDK4/6 inhibitors (palbociclib, ribociclib, abemaciclib)	Enhances endocrine therapy efficacy in HR+ early-onset breast cancers

### Clinical implications

The unique biological and molecular characteristics of breast cancer in young women have profound clinical implications that necessitate tailored diagnostic, therapeutic, and survivorship strategies. Recognizing these differences is critical for optimizing patient outcomes and addressing the distinct challenges faced by this population^[^29^]^. From a diagnostic perspective, the aggressive nature of tumors in young women underscores the need for heightened clinical vigilance and earlier detection approaches. Conventional screening guidelines, which primarily target older women, may not adequately identify high-risk younger patients. Incorporating genetic testing – especially for BRCA1/2 and other hereditary cancer predisposition genes – is crucial for risk stratification and guiding preventive measures. Furthermore, molecular profiling of tumors should be routinely performed to accurately classify intrinsic subtypes and identify actionable targets, thereby informing personalized treatment plans^[^30^]^.

**Figure 1.**
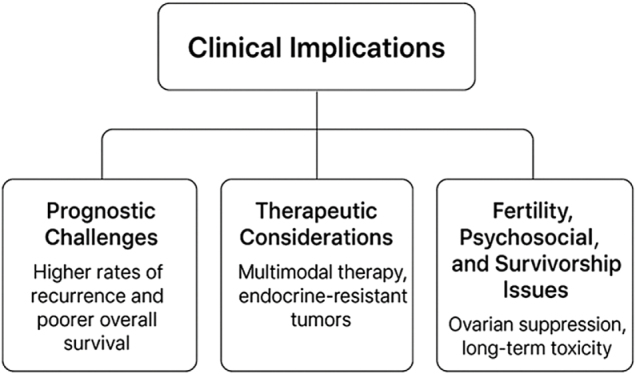
Clinical implications.

Therapeutically, young women with breast cancer often require more intensive and multimodal treatment regimens due to the predominance of aggressive subtypes such as triple-negative and HER2-positive cancers. Standard treatments including surgery, chemotherapy, radiotherapy, and targeted agents must be carefully balanced against potential long-term toxicities that disproportionately affect younger patients. Fertility preservation, management of treatment-induced premature menopause, and psychosocial support are vital considerations that must be integrated into comprehensive care plans^[^31^]^. The emerging role of targeted therapies, such as PARP inhibitors for BRCA-mutated tumors and CDK4/6 inhibitors for hormone receptor-positive disease, highlights the importance of molecularly guided treatment. Immune checkpoint inhibitors have shown promise, particularly in TNBC, but response rates remain variable, underscoring the need for predictive biomarkers and combination approaches tailored to the young patient’s tumor microenvironment^[^32^]^. Survivorship care also requires specialized attention. Young breast cancer survivors face unique challenges including higher risks of recurrence, secondary malignancies, and treatment-related comorbidities. Addressing quality of life issues such as fertility, sexual health, bone density preservation, and psychosocial well-being is critical for holistic patient management. Multidisciplinary care teams should coordinate long-term follow-up that encompasses both oncologic and supportive care needs (Fig. 1)^[^33,34,35^]^.

## Conclusion

Breast cancer in young women represents a biologically distinct and clinically challenging subset of the disease, characterized by aggressive tumor phenotypes, unique molecular alterations, and a complex tumor microenvironment. The distinct hormone receptor and HER2 expression patterns, coupled with specific genomic and epigenomic signatures, underscore the need for age-tailored diagnostic and therapeutic approaches. These molecular insights have unveiled critical pathways driving tumor progression and resistance, offering promising avenues for targeted interventions. Advances in understanding the interplay between tumor biology and the immune landscape highlight opportunities to leverage immunotherapy and stroma-modulating strategies in young patients. However, the heterogeneity within this population demands further research to develop predictive biomarkers and optimize personalized treatment regimens. Clinically, integrating molecular profiling into routine care is essential for accurate risk stratification and treatment selection, while addressing the unique psychosocial and reproductive health needs of young women is paramount for comprehensive survivorship care.

## Data Availability

The data that support the findings of this study are available from the corresponding author upon reasonable request.

## References

[1] Łukasiewicz S Czeczelewski M Forma A. Breast cancer-epidemiology, risk factors, classification, prognostic markers, and current treatment strategies-an updated review. Cancers (Basel) 2021;13:4287. 34503097 10.3390/cancers13174287 PMC8428369

[2] Roheel A Khan A Anwar F. Global epidemiology of breast cancer based on risk factors: a systematic review. Front Oncol 2023;13:1240098. 37886170 10.3389/fonc.2023.1240098 PMC10598331

[3] Stibbards-Lyle M Malinovska J Badawy S. Status of breast cancer detection in young women and potential of liquid biopsy. Front Oncol 2024;14:1398196. 38835377 10.3389/fonc.2024.1398196 PMC11148378

[4] Erić I Petek Erić A Kristek J. Breast cancer in young women: pathologic and immunohistochemical features. Acta Clin Croat 2018;57:497–502. 31168183 10.20471/acc.2018.57.03.13 PMC6536281

[5] Srikanthan A Awan AA McGee S. Young women with breast cancer: the current role of precision oncology. J Pers Med 2023;13:1620. 38003935 10.3390/jpm13111620 PMC10672565

[6] Agha RA Mathew G Rashid R. TITAN Group. Transparency in the reporting of artificial intelligence – the TITAN Guideline. Prem J Sci 2025;10:100082.

[7] Deshmukh SK Srivastava SK Tyagi N. Emerging evidence for the role of differential tumor microenvironment in breast cancer racial disparity: a closer look at the surroundings. Carcinogenesis 2017;38:757–65. 28430867 10.1093/carcin/bgx037 PMC5862302

[8] Vaz-Luis I Ottesen RA Hughes ME. Impact of hormone receptor status on patterns of recurrence and clinical outcomes among patients with human epidermal growth factor-2-positive breast cancer in the National Comprehensive Cancer Network: a prospective cohort study. Breast Cancer Res 2012;14:R129. 23025714 10.1186/bcr3324 PMC4053106

[9] Walbaum B Martínez-Sáez O Brasó-Maristany F. Genomic and clinical features in young women with estrogen receptor-positive, HER2-negative breast cancer. ESMO Open 2025;10:105764. 40997745 10.1016/j.esmoop.2025.105764 PMC12495081

[10] Pérez-López ME García-Gómez J Alves MT. Ki-67 is a prognostic marker for hormone receptor positive tumors. Clin Transl Oncol 2016;18:996–1002. 26742937 10.1007/s12094-015-1472-y PMC5018017

[11] Cheng X. A comprehensive review of HER2 in cancer biology and therapeutics. Genes (Basel) 2024;15:903. 39062682 10.3390/genes15070903 PMC11275319

[12] Dix-Peek T Phakathi BP van den Berg EJ. Discordance between PAM50 intrinsic subtyping and immunohistochemistry in South African women with breast cancer. Breast Cancer Res Treat 2023;199:1–12. 36867282 10.1007/s10549-023-06886-3 PMC10147771

[13] Nikita N Sun Z Sharma S. Epigenetic landscapes of aging in breast cancer survivors: unraveling the impact of therapeutic interventions-a scoping review. Cancers (Basel) 2025;17:866. 40075712 10.3390/cancers17050866 PMC11899678

[14] Godet I Gilkes DM. BRCA1 and BRCA2 mutations and treatment strategies for breast cancer. Integr Cancer Sci Ther 2017;4:10.15761/ICST.1000228. 10.15761/ICST.1000228 PMC5505673 28706734

[15] Bediaga NG Acha-Sagredo A Guerra I. DNA methylation epigenotypes in breast cancer molecular subtypes. Breast Cancer Res 2010;12:R77. 20920229 10.1186/bcr2721 PMC3096970

[16] Bure IV Nemtsova MV Kuznetsova EB. Histone modifications and non-coding RNAs: mutual epigenetic regulation and role in pathogenesis. Int J Mol Sci 2022;23:5801. 35628612 10.3390/ijms23105801 PMC9146199

[17] Wang ZZ Li XH Wen XL. Integration of multi-omics data reveals a novel hybrid breast cancer subtype and its biomarkers. Front Oncol 2023;13:1130092. 37064087 10.3389/fonc.2023.1130092 PMC10091394

[18] Li JJ Tsang JY Tse GM. Tumor microenvironment in breast cancer-updates on therapeutic implications and pathologic assessment. Cancers (Basel) 2021;13:4233. 34439387 10.3390/cancers13164233 PMC8394502

[19] Valenza C Taurelli Salimbeni B Santoro C. Tumor infiltrating lymphocytes across breast cancer subtypes: current issues for biomarker assessment. Cancers (Basel) 2023;15:767. 36765724 10.3390/cancers15030767 PMC9913599

[20] Huertas-Caro CA Ramírez MA Rey-Vargas L. Tumor infiltrating lymphocytes (TILs) are a prognosis biomarker in Colombian patients with triple negative breast cancer. Sci Rep 2023;13:21324. 38044375 10.1038/s41598-023-48300-4 PMC10694133

[21] Zhou Z Zhong H Wang H. Microenvironmental regulation and remodeling of breast cancer angiogenesis: from basic mechanisms to clinical therapeutic implications. Discov Oncol 2025;16:1973. 41144119 10.1007/s12672-025-03797-1 PMC12559528

[22] Abdul-Rahman T Ghosh S Badar SM. The paradoxical role of cytokines and chemokines at the tumor microenvironment: a comprehensive review. Eur J Med Res 2024;29:124. 38360737 10.1186/s40001-024-01711-z PMC10868116

[23] Miglietta F Griguolo G Guarneri V. Programmed cell death ligand 1 in breast cancer: technical aspects, prognostic implications, and predictive value. Oncologist 2019;24:e1055–e1069. 31444294 10.1634/theoncologist.2019-0197 PMC6853089

[24] Maqsood Q Khan MU Fatima T. Recent insights into breast cancer: molecular pathways, epigenetic regulation, and emerging targeted therapies. Breast Cancer (Auckl) 2025;19:11782234251355663. 40661160 10.1177/11782234251355663 PMC12256763

[25] Li H Prever L Hirsch E. Targeting PI3K/AKT/mTOR Signaling Pathway in Breast Cancer. Cancers (Basel) 2021;13:3517. 34298731 10.3390/cancers13143517 PMC8304822

[26] Li J Jia Z Dong L. DNA damage response in breast cancer and its significant role in guiding novel precise therapies. Biomark Res 2024;12:111. 39334297 10.1186/s40364-024-00653-2 PMC11437670

[27] Iqbal N Iqbal N. Human epidermal growth factor receptor 2 (HER2) in cancers: overexpression and therapeutic implications. Mol Biol Int 2014;2014:852748. 25276427 10.1155/2014/852748 PMC4170925

[28] Ryspayeva D Seyhan AA MacDonald WJ. Signaling pathway dysregulation in breast cancer. Oncotarget 2025;16:168–201. 40080721 10.18632/oncotarget.28701 PMC11906143

[29] Yang X Yang D Qi X. Endocrine treatment mechanisms in triple-positive breast cancer: from targeted therapies to advances in precision medicine. Front Oncol 2025;14:1467033. 39845328 10.3389/fonc.2024.1467033 PMC11753220

[30] Colleoni M Anders CK. Debate: the biology of breast cancer in young women is unique. Oncologist 2013;18:e13–5. 23633450 10.1634/theoncologist.2013-0118 PMC3639538

[31] Nasim Z Girtain C Gupta V. Breast cancer incidence and behavior in younger patients: a study from the surveillance, epidemiology and end results database. World J Oncol 2020;11:88–97. 32494315 10.14740/wjon1278 PMC7239572

[32] Obidiro O Battogtokh G Akala EO. Triple negative breast cancer treatment options and limitations: future outlook. Pharmaceutics 2023;15:1796. 37513983 10.3390/pharmaceutics15071796 PMC10384267

[33] Howard FM Villamar D He G. The emerging role of immune checkpoint inhibitors for the treatment of breast cancer. Expert Opin Investig Drugs 2022;31:531–48. 10.1080/13543784.2022.1986002 PMC8995399 34569400

[34] Bodai BI Tuso P. Breast cancer survivorship: a comprehensive review of long-term medical issues and lifestyle recommendations. Perm J 2015;19:48–79. 10.7812/TPP/14-241 PMC4403581 25902343

[35] Suba Z. Triple-negative breast cancer risk in women is defined by the defect of estrogen signaling: preventive and therapeutic implications. Onco Targets Ther 2014;7:147–64. 24482576 10.2147/OTT.S52600 PMC3905095

